# Qualitative study on the factors leading to variation in experience of the Foundation Psychiatry Fellowship of the Royal College of Psychiatrists

**DOI:** 10.1192/j.eurpsy.2023.248

**Published:** 2023-07-19

**Authors:** K. Denediou Derrer

**Affiliations:** Hertfordshire Partnership Foundation Trust, Hatfield; Higher Education England, East of England, Cambridge, United Kingdom

## Abstract

**Introduction:**

The Psychiatry Foundation Fellowships were created by the Royal College of Psychiatrists (RCPsych) as a route to encourage foundation doctors to consider psychiatry as an exciting medical discipline.

**Objectives:**

This study aimed to explore the Psychiatry Foundation Fellows’ experience of applying to the Fellowship, their expectations prior to being appointed, the benefits of the fellowship, the barriers to gaining those benefits, any common factors raised, and any suggestions about how to improve the fellowship.

**Methods:**

The researcher was a leadership fellow in medical education and simulation in the Foundation school of East of England. Ethical approval was obtained through Higher Education England as this was a service evaluation. Recruitment was purposive and participants were contacted by a gatekeeper. Four 1:1 interviews took place, the interviews were audio recorded, transcribed and the transcripts were analysed with thematic analysis.

**Results:**

Preliminary Themes

**Table:** 

Opportunities/ Facilitators	Pursuit of Psychiatry-related opportunities; Study leave budget for Psychiatry-related courses Balint group while in psychiatry rotation Guaranteed psychiatry rotation	
Barriers	Psychiatry Fellowship supervision	-Location of supervisor -Frequency of supervision -Supervisors’ availability to meet at a time convenient for fellow rather than on a fellow’s day off -Allocation of supervisors who were willing to act as a mentor, who had suggestions for projects and were committed to stay in the role for the 2 years.
	Social connection with other psychiatry foundation fellows	-Data protection rules -Lack of community -40 fellows spread across the UK -Fellow’s rotas preventing them from pursuing social connections -Lack of Balint group with other Foundation fellows
	Identity as a Psychiatry foundation fellow	-Lack of recognition / awareness by clinical supervisors -Difficult to take advantage of the opportunities of the fellowship -Fellow needing to advocate for oneself -Balint group of psychiatry rotations was usually for core psychiatry trainees -Rota coordinators gatekeepers for study leave
	Identity as a foundation doctor	-Timing of the psychiatry rotation -Feel “out of the loop” compared to peers when entering acute hospitals after psychiatry rotation, if it is first in FY1
	Impact of Covid	-Intense medical rotations, poorly staffed rotas -All non-departmental teaching was suspended, which made it even more difficult to justify self-development -One social opportunity per year only, to meet other Foundation fellows

**Conclusions:**

The Psychiatry Foundation Fellowship was generally a positive experience in terms of fostering enthusiasm for psychiatry. A sense of community among fellows and recognition among clinical supervisors in acute trusts were felt to be lacking. The themes were used to shape RCPsych’s future plans for the Psychiatry Foundation Fellowship.

**Disclosure of Interest:**

None Declared

